# Supplementary material for: Epicardial conduction abnormalities in patients with Arrhythmogenic Right Ventricular Cardiomyopathy (ARVC) and mutation positive healthy family members – A study using electrocardiographic imaging
Source: PLoS One. 2023 Jan 5;18(1):e0280111. doi: 10.1371/journal.pone.0280111 (PMC9815642; doi:10.1371/journal.pone.0280111)
Supplement: S1 Table — The table summarizes the main findings of the study regarding repolarization and depolarization and compares them with the findings from a 12-lead ECG. (Tw: T-wave, TwI: T-wave inversion, EW: epsilon-wave, TAD: terminal activation duration, LP: late potential, AW: anterior wall, IWa: apical segment of inferior wall, IWb: basal segment of inferior wall, P: positive, N: negative, Iso: isoelectric, Biph: biphasic, TA: terminal activation, ms: milliseconds). *Refers to the number of LP criteria that are fulfilled. (DOCX) [file pone.0280111.s001.docx]

S1 Table. Summary of the repolarization and depolarization characteristics of the cohort

|  |  | **ECG** | | | | | | **Epicardial signals/ ECGI** | | | | | |
| --- | --- | --- | --- | --- | --- | --- | --- | --- | --- | --- | --- | --- | --- |
|  | **ID** | **TwI**  **V1-V3** | **TwI**  **V5-V6, I, aVL** | **TwI**  **II, III, aVF** | **EW** | **TAD >55ms** | **LP* criteria** | **Tw**  **AW** | **Tw**  **RVOT** | **Tw**  **base** | **Tw**  **IWa** | **Tw**  **IWb** | **Abnormal TA** |
| **Controls** | C1 | **-** | **-** | **-** | **-** | **-** | 1 | **P** | **P** | **N** | **P** | **P** | **-** |
|  | C2 | **-** | **-** | **-** | **-** | **-** | 0 | **P** | **N** | **Iso** | **P** | **P** | **-** |
|  | C3 | **-** | **-** | **-** | **-** | **-** | 0 | **P** | **P** | **Iso** | **P** | **P** | **-** |
|  | C4 | **-** | **-** | **-** | **-** | **-** | 0 | **N** | **P** | **N** | **N** | **N** | **-** |
|  | C5 | **-** | **-** | **-** | **-** | **-** | 0 | **P** | **P** | **P** | **P** | **P** | **-** |
|  | C6 | **-** | **-** | **-** | **-** | **-** | 0 | **P** | **P** | **P** | **P** | **P** | **-** |
|  | C7 | **-** | **-** | **-** | **-** | **-** | 0 | **P** | **P** | **N** | **Iso** | **N** | **-** |
|  | C8 | **-** | **-** | **-** | **-** | **-** | 1 | **Iso** | **N** | **Iso** | **N** | **N** | **-** |
| **ARVC patients** | P1 | **-** | **-** | **+** | **-** | **-** | 3 | **P** | **P** | **Iso** | **N** | **N** | **+** |
|  | P2 | **+** | **-** | **-** | **-** | **+** | 3 | **N** | **N** | **N** | **N** | **N** | **+** |
|  | P3 | **+** | **+** | **-** | **-** | **+** | 3 | **N** | **N** | **N** | **N** | **N** | **+** |
|  | P4 | **+** | **+** | **-** | **+** | **+** | 3 | **N** | **N** | **Iso** | **N** | **N** | **+** |
|  | P5 | **+** | **-** | **+** | **-** | **+** | 3 | **N** | **N** | **N** | **N** | **N** | **+** |
|  | P6 | **+** | **+** | **+** | **+** | **+** | 3 | **N** | **N** | **N** | **N** | **N** | **+** |
|  | P7 | **+** | **-** | **+** | **-** | **-** | 3 | **N** | **Biph** | **N** | **N** | **N** | **+** |
|  | P8 | **+** | **+** | **-** | **+** | **+** | 3 | **Iso** | **N** | **Iso** | **Iso** | **Iso** | **+** |
|  | P9 | **+** | **+** | **+** | **+** | **-** | 3 | **N** | **N** | **N** | **N** | **N** | **+** |
|  | P10 | **+** | **+** | **-** | **+** | **+** | 3 | **Biph** | **Biph** | **Biph** | **N** | **N** | **+** |
|  | P11 | **+** | **-** | **+** | **-** | **-** | 0 | **Iso** | **N** | **N** | **N** | **N** | **+** |
|  | P12 | **-** | **-** | **+** | **+** | **+** | 3 | **Iso** | **N** | **Iso** | **N** | **N** | **+** |
| **Genotype-positive phenotype-negative subjects** | G1 | **-** | **-** | **-** | **-** | **-** | 0 | **P** | **P** | **P** | **P** | **P** | **+** |
|  | G2 | **-** | **-** | **-** | **-** | **-** | 0 | **P** | **P** | **Iso** | **Iso** | **N** | **+** |
|  | G3 | **-** | **-** | **-** | **-** | **-** | 0 | **P** | **P** | **N** | **N** | **N** | **-** |
|  | G4 | **-** | **-** | **-** | **-** | **-** | 3 | **P** | **P** | **P** | **P** | **N** | **+** |
|  | G5 | **-** | **-** | **-** | **-** | **-** | 0 | **P** | **P** | **N** | **P** | **N** | **-** |
|  | G6 | **-** | **-** | **-** | **-** | **-** | 0 | **P** | **P** | **P** | **P** | **P** | **+** |
|  | G7 | **-** | **-** | **-** | **-** | **-** | 3 | **N** | **N** | **N** | **N** | **N** | **+** |
|  | G8 | **-** | **-** | **-** | **-** | **-** | 0 | **N** | **Iso** | **N** | **N** | **N** | **-** |
|  | G9 | **+** | **-** | **-** | **-** | **-** | 0 | **N** | **Iso** | **N** | **N** | **N** | **-** |
|  | G10 | **-** | **-** | **-** | **-** | **+** | 3 | **P** | **P** | **N** | **P** | **N** | **+** |
|  | G11 | **-** | **-** | **-** | **-** | **-** | 0 | **Iso** | **Iso** | **Iso** | **N** | **N** | **+** |
|  | G12 | **-** | **-** | **-** | **-** | **+** | 1 | **P** | **N** | **N** | **N** | **N** | **-** |
|  | G13 | **-** | **-** | **-** | **-** | **-** | 0 | **P** | **P** | **N** | **N** | **N** | **-** |
|  | G14 | **-** | **-** | **-** | **-** | **-** | 0 | **P** | **P** | **P** | **N** | **N** | **+** |
|  | G15 | **-** | **-** | **-** | **-** | **-** | 0 | **P** | **N** | **N** | **N** | **N** | **+** |
|  | G16 | **-** | **-** | **-** | **-** | **-** | 0 | **P** | **P** | **Iso** | **P** | **P** | **-** |
|  | G17 | **-** | **-** | **-** | **-** | **-** | 0 | **P** | **P** | **P** | **P** | **P** | **-** |
|  | G18 | **-** | **-** | **-** | **-** | **-** | 1 | **Iso** | **Iso** | **Iso** | **Iso** | **Iso** | **-** |
|  | G19 | **+** | **-** | **-** | **-** | **-** | 0 | **N** | **N** | **N** | **N** | **N** | **-** |
|  | G20 | **-** | **-** | **-** |  |  |  | **Iso** | **Iso** | **N** | **N** | **N** | **+** |

The table summarizes the main findings of the study regarding repolarization and depolarization and compares them with the findings from a 12-lead ECG.

(Tw: T-wave, TwI: T-wave inversion, EW: epsilon-wave, TAD: terminal activation duration, LP: late potential, AW: anterior wall, IWa: apical segment of inferior wall, IWb: basal segment of inferior wall, P: positive, N: negative, Iso: isoelectric, Biph: biphasic, TA: terminal activation, ms: milliseconds)

*Refers to the number of LP criteria that are fulfilled.
